# Validation of the CaReQoL asthma: a patient reported outcome measure for monitoring the perceived effects of pulmonary rehabilitation in adult patients with severe refractory asthma

**DOI:** 10.1186/s12931-022-02281-6

**Published:** 2023-01-13

**Authors:** Linda Springvloet, Mattanja Triemstra, Bart Knottnerus, Marlon Rolink, Harry Heijerman, Dolf de Boer

**Affiliations:** 1 Nivel, Netherlands Institute for Health Services Research, PO Box 1568, 3500 BN Utrecht, The Netherlands; 2 Department of Respiratory Medicine, University Medical Center Utrecht, Utrecht, The Netherlands

**Keywords:** Asthma, Pulmonary rehabilitation, Patient reported outcome measurement (PROM), Validity, Responsiveness

## Abstract

**Background:**

The CaReQoL Asthma assesses the care-related quality of life outcomes of pulmonary rehabilitation retrospectively in patients with severe asthma. The questionnaire comprises five domains (physical functioning; social functioning; coping with asthma; knowledge about asthma; medication).

**Aim:**

To investigate construct and criterion validity of the CaReQoL Asthma, as well as its responsiveness and minimal important change (MIC), in comparison with other health measures (AQLQ, ACQ and FEV_1_).

**Methods:**

Eighty three adults with severe refractory asthma filled out the CaReQoL Asthma at 6 and 12 months after a 12-week personalized multidisciplinary pulmonary rehabilitation program in a tertiary asthma centre, either in Switzerland or The Netherlands. Construct validity and responsiveness were assessed by testing pre-defined hypotheses about associations with changes in AQLQ, ACQ and FEV_1_ scores. Criterion validity and MIC was assessed using Global Perceived Effect (GPE). Factor analyses, Cronbach’s alpha, Spearman's correlations, paired t-tests and Student–Newman–Keuls tests were performed.

**Results:**

Cronbach’s alphas of the questionnaire domains ranged from 0.82 to 0.95. Good construct validity and responsiveness were found; 84% of the assessed correlations confirm pre-defined hypotheses and reflect both weak and moderate to strong correlations. Good criterion validity was also identified, with CaReQol scores discriminating better than other health measures between levels of GPE at 6 months post-rehabilitation. The MIC for the total score was estimated at 0.84.

**Conclusion:**

These study results suggest that the CaReQoL Asthma is a valid and responsive instrument and shows to be a comprehensive and tailored questionnaire for evaluating and monitoring outcomes of pulmonary rehabilitation in patients with severe refractory asthma. In order to further substantiate the reliability and validity of the CaReQoL Asthma, as well as to monitor outcomes of pulmonary rehabilitation in patients with severe asthma, it is recommended to use the CaReQoL Asthma in addition to other disease specific instruments.

## Background

Severe refractory asthma is asthma that remains uncontrolled despite optimal pharmacological treatment with high dose inhaled corticosteroids plus another controller and/or systemic corticosteroids, and adequately addressing comorbidities and contributory factors [1]. This chronic condition and its treatment poses a substantial burden on the patients’ life, due to the symptoms, exacerbations and side-effects of the medication, with profound consequences for the patients’ physical and mental health, relationships and careers [2]. In the Netherlands, approximately 3.6% of the adults with asthma have severe refractory asthma and their care is estimated to account for more than 60% of the costs associated with asthma [1, 3].

Besides pharmacological treatment, non-pharmacological interventions such as allergen avoidance and pulmonary rehabilitation are recommended for severe refractory asthma [4, 5]. A pulmonary rehabilitation trajectory is often indicated. Rehabilitation is the process of helping a person achieve the highest possible level of function, independence and quality of life. Pulmonary rehabilitation is defined as a comprehensive intervention based on a thorough patient assessment followed by patient-tailored therapies, which include but are not limited to exercise training, education, self-management and psychological support, designed to improve the physical and psychological condition of patients with chronic respiratory disease and to promote the long-term adherence of health-enhancing behaviors [6]. During and after this trajectory, effects can be monitored by regularly measuring the lung function (e.g. forced expiratory volume in one second, FEV_1_) and patient-reported outcome measures (PROMs; i.e. questionnaires on the self-perceived health status or health-related quality of life) [7, 8].

PROMs are frequently used in asthma care, for example to assess the perceived severity or health status and quality of life of patients. Two internationally well-known, validated and frequently used instruments are the Asthma Quality of Life Questionnaire (AQLQ) [9, 10] and the Asthma Control Questionnaire (ACQ) [11]. However, these are not specifically designed for assessing the effects of pulmonary rehabilitation and might fall short in fully describing the health-related quality of life from the patients’ perspective with respect to the treatment goals. In general, there is a need for more comprehensive assessments to identify treatable traits that can be addressed during rehabilitation [12]. But until now, specific instruments for comprehensively assessing the outcomes of multidisciplinary pulmonary rehabilitation for patients with severe asthma are missing.

Therefore, we have developed the CaReQoL Asthma to measure the Care-Related Quality of Life; a disease and care-specific questionnaire for monitoring the effects of clinical rehabilitation in patients with severe asthma [13]. Patients were fully involved in all stages of the development, in contrast to many previously developed PROMs (e.g. the ACQ) that have been developed by clinicians or researchers and consequently do not necessarily represent the patients’ perspective [14]. The development process of the CaReQoL Asthma is summarized in Box 1. The items of the questionnaire were based on focus groups with patients, literature and existing questionnaires, and extensive cognitive and psychometric testing in patients [13].

**Box 1:**
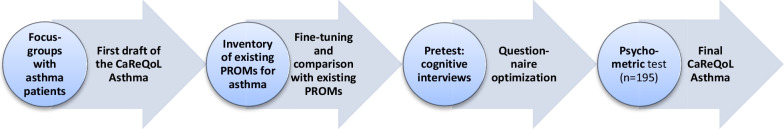
Development process of the CaReQoL Asthma (Van Kessel et al. [13])

This study aims to assess the construct and criterion validity of the CaReQoL Asthma, by comparing the scores with two other self-report asthma-specific health questionnaires (ACQ and AQLQ) and a clinical measure (FEV_1_), and to assess the responsiveness and the minimal important change (MIC) of the CaReQoL Asthma. We hypothesize that this care-specific instrument assesses the care-related health outcomes of asthma rehabilitation more comprehensively and specifically than other asthma-specific questionnaires (ACQ and AQLQ), and that the CaReQoL Asthma is a more tailored, straightforward, and responsive instrument for assessing the outcomes of asthma rehabilitation longitudinally than the other instruments and the clinical measure for lung function (predicted FEV_1_).

## Methods

### Study design and treatment setting

Data were collected as an additional, informal part of a prospective clinical study with a 12 months follow-up period on the effects of a 12-week personalized multidisciplinary pulmonary rehabilitation program, either at a high-altitude (Dutch Asthma Centre in Davos, Switzerland) or at sea-level (Merem Asthma Centre in Hilversum, The Netherlands) [15]. The clinical study was originally set up as a trial and was registered at The Netherlands Trial Register (www.trialregister.nl; NTR5182), but it fell back to an observational design because randomization turned out to be not feasible. Nevertheless, patients were assessed and evaluated during and after their pulmonary rehabilitation in accordance with a systematic protocol.

Both asthma centres supplied structured, quality-controlled personalized treatment and pulmonary rehabilitation for adults with severe asthma. The rehabilitation included attempts to achieve optimal asthma control and to reduce (oral) corticosteroids to the lowest effective level, exercise training, asthma education including self-management, and psychological support. Both centres provided standardized treatment by using a modular approach with nine basic modules: medication and inhalation; exacerbation; self-management; physical fitness; daily physical activity; functional-ADL-training, dyspnea management; food and diet; coping; psychological support.

After the 12-week rehabilitation period, patients were followed for an additional 12 months with follow-up visits every 3 months at the asthma centre in The Netherlands. During the follow-up, patients were treated by their referring pulmonologist according to the (international) guidelines.

### Patients

This validation study includes 83 adults (18–75 years) with severe refractory asthma who were living in The Netherlands and who filled out the CaReQoL Asthma at follow-up at 6 and 12 months after the pulmonary rehabilitation. All selected patients had a diagnosis of severe refractory asthma according to the ERS/ATS criteria [1], and they were referred by their pulmonologist to a tertiary asthma clinic between October 2015 and February 2018 [15].

At baseline, all patients were symptomatic and had uncontrolled asthma, they used long-acting bronchodilators and high dose inhaled corticosteroids with or without oral corticosteroid, and they were either non-smokers or ex-smokers for > 6 months [15]. Treatment of comorbidity was optimized before taking part in the study [15]. Uncontrolled asthma was defined by the presence of at least two of the following criteria: (1) poor symptom control defined as an ACQ-score ≥ 1.5 or an ACT-score < 20; (2) frequent severe exacerbations defined as two or more bursts of systemic corticosteroids (> 3 days) in the previous year; (3) serious exacerbations defined as at least one hospitalization or intensive care unit stay or mechanical ventilation in the previous year because of an asthma exacerbation; and/or (4) persistent airflow limitation (post-bronchodilator FEV1 < 80% predicted or a FEV1/forced vital capacity (FCV) z-score < 1.64). More details on the recruitment and selection procedure and the exclusion criteria are described in a previous paper on the clinical study by De Nijs et al. [15].

Patients voluntarily completed the CaReQoL Asthma in addition to the primary and secondary outcome measures of the clinical study. All patients provided their written informed consent on taking part in the clinical study which was approved by the Ethics Committee of the Academic Medical Center of the University of Amsterdam (Amsterdam, The Netherlands) [15]. The CaReQoL Asthma was not officially part of the clinical study, and an amendment about the additional informal data collection with this instrument was handed over to the Ethics Committee after the start of the clinical study.

### Data collection and instruments

Data collection with the CaReQoL Asthma was an additional, informal part of the clinical study. Measurement points of the clinical study itself were at baseline or entry of the study (t0) and two additional follow-up measurements after completing the 12-week pulmonary rehabilitation trajectory: after 6 months (38 weeks after entry; t38) and at 12 months (64 weeks after entry; t64).

Apart from the CaReQoL Asthma, two other asthma-specific self-report questionnaires were used: the Asthma Quality of Life Questionnaire (AQLQ) and Asthma Control Questionnaire (ACQ), and a clinical measure for lung functioning (FEV_1_). The CaReQoL Asthma was only assessed retrospectively at t38 and t64 (i.e. 38 and 64 weeks after the start of the rehabilitation), whereas the other instruments (AQLQ, ACQ) and the FEV_1_ were assessed at all three points including baseline (t0, t38 and t64).

#### CaReQoL asthma

The Care Related Quality of Life in Asthma (CaReQoL Asthma) is a 26-item questionnaire about the perceived effects of pulmonary rehabilitation on the quality of life of asthma patients [13]. This PROM aims to retrospectively evaluate the ‘Care Related Quality of Life’, by asking asthma patients directly to rate the effects of rehabilitation on various health aspects and their overall quality of life. The face and content validity of this questionnaire have already been pre-tested in 15 cognitive interviews and a psychometric test based on a survey among 195 patients [13]. See Appendix for the content of the questionnaire.

The CaReQoL Asthma comprises five domains or scales: Physical functioning (8 items), Social functioning (4 items), Coping with asthma (5 items), Knowledge about asthma (3 items), and Medication (3 items). The 23 items measuring these five domains state to what extent the pulmonary rehabilitation contributed to the patients’ treatment goals and health aspects, with a 5-point answering scale: 1 = completely disagree, 2 = partly disagree, 3 = neutral (not agree, nor disagree), 4 = partly agree, 5 = completely agree. The escape option ‘not applicable’ was coded as missing. For each domain, an average scale score was calculated from the item scores (range 1–5), only for patients who completed more than half of the items within the corresponding domain. All five scales previously showed to have good internal consistency, with Cronbach’s alpha’s between 0.75 and 0.96 [13].

The last three items of the CaReQoL Asthma reflect on the overall effect of pulmonary rehabilitation on the quality of life (1 item; 1 = completely disagree, to 5 = completely agree), the global perceived effect (GPE [16]) of the rehabilitation on the patient’s health (1 item (How is your health, compared to your health prior to rehabilitation?); 1 = very much deterioration, 2 = much deterioration; 3 = little deterioration; 4 = no change; 5 = little improvement; 6 = much improvement; 7 = very much improvement), and the respondent’s age (in years), respectively.

#### Asthma quality of life questionnaire

The Asthma Quality of Life Questionnaire (AQLQ) is an asthma-specific 32-item questionnaire that measures the health-related quality of life of adults with asthma for four domains: Symptoms; Activity limitations; Emotional functioning; and Environmental stimuli [9]. Four domain scores and a total score were calculated (means of all item-scores). The Minimal Important Difference (MID) for the AQLQ is 0.5, which means that a change in score of 0.5 on the 7-point scale is the smallest change that can be considered clinically important [17].

#### Asthma control questionnaire

The Asthma Control Questionnaire (ACQ) measures both the adequacy of asthma control and change in asthma control, which occurs either spontaneously or as a result of treatment. In this study, a 6-item version of the ACQ questionnaire was used (without the clinic staff score of the FEV_1_ predicted) [18]. The ACQ score was calculated as the mean of the 6 symptom-related items (0 = totally controlled; 6 = severely uncontrolled; with scores < 0.5 indicating ‘controlled asthma’ and > 1.5 ‘uncontrolled asthma’). A change or difference in ACQ score of at least 0.5 can be considered as clinically important [18].

#### Forced expiratory volume in one second

Pulmonary function was measured according to international recommendations and included the forced vital capacity (FVC) and the forced expiratory volume in one second (FEV_1_), assessed after inhaled administration of 400 μg salbutamol and expressed as percentage of the predicted value [19]. The FEV_1_ at baseline (t0), t38 and t64 was used as an indicator of the pulmonary function in this paper.

### Statistical analyses

The analyses focused on the construct validity (factor structure, internal consistency, convergent and divergent validity), criterion validity, responsiveness and the minimal important change (MIC). Analyses included: factor analyses, internal consistency (Cronbach’s alpha), Spearman's rank correlation coefficients (r), mean scores and 95% confidence intervals, paired t-tests and Student–Newman–Keuls (SNK) tests. All analyses were performed in Stata/SE (version 15) and a p-value of < 0.05 was considered as statistically significant.

Since the response rate for the CaReQoL Asthma was the highest at t38, this measurement point was mainly used to investigate the psychometric properties of the CaReQoL Asthma and to compare the CaReQol scores with change scores of the AQLQ, ACQ and FEV_1_ (∆ t0–t38). Except for assessing the responsiveness, for which the scores at t64 were used as well.

#### Construct validity

Defining the five domains of the CaReQoL Asthma was based on psychometric analyses, including factor analyses, conducted in a previous study (factor loadings range: 0.35–0.93, inter-scale correlations range: 0.59–0.90, n = 195) [13]. To confirm the psychometric properties of the questionnaire in the current study sample, factor analyses were conducted for each subscale separately (Principal Component Analyses, with oblimin rotations; Eigenvalue > 1 and factor loadings > 0.40). Then the internal consistency of the subscales was assessed by calculating the Cronbach’s alpha for each scale. An alpha of > 0.70 is generally considered as a good reliability [8, 20].

Subsequently, Spearman’s rank correlation coefficients (r) were calculated to see whether the scales or items of the CaReQoL Asthma do significantly correlate with other similar measures (i.e. convergent validity), or do not (i.e. divergent or discriminant validity). Convergent/divergent validity was assessed by calculating Spearman’s rank correlations between CaReQoL Asthma scores (t38) and change scores (Δ t0–t38) of similar domains of the AQLQ and the ACQ.

Based on the questionnaires’ content and more or less similar items, moderate correlations (r = 0.40–0.59) were expected between the following CaReQoL scores and related domains or items in the AQLQ and ACQ questionnaires:

- Physical functioning vs. changes in AQLQ Total score, AQLQ Symptoms, AQLQ Activity limitations, and ACQ;
- Social functioning vs. changes in AQLQ Total score, AQLQ Activity limitations and ACQ;
- Coping with asthma vs. changes in AQLQ Emotional functioning and ACQ;
- Medication vs. changes in AQLQ Symptoms and ACQ;
- Overall quality of life (1 item) vs. changes in AQLQ Total score, AQLQ Symptoms and ACQ;
- Global perceived effect (1 item) vs. changes in AQLQ Total score, AQLQ Symptoms and ACQ.

Weak (r < 0.40) or insignificant (p > 0.05) correlations were expected between all other combinations of total and domain scores, which would confirm divergent validity. See Table 3 for the hypothesized relationships. Good construct validity means that at least 75% of the hypotheses are correct [20].

#### Criterion validity

Criterion validity refers to a comparison between the measure in question and an outcome assessed at the same time (concurrent validity), and the extent to which the measure is related to or predicts a concrete outcome or criterion (predictive validity) [20]. In this study the GPE (global perceived effect) was chosen as a criterion, because it is a concrete and global measure for the perceived effect of the pulmonary rehabilitation at t38. The criterion validity was assessed by first subdividing respondents into three groups based on the self-reported global perceived effect (GPE) at t38, and by comparing the three subgroups with respect to the five CaReQoL domain scores and the change scores of the other health measures since the start of the rehabilitation trajectory (Δ t0–t38).

The three subgroups according to the criterion variable (GPE) are categorized as follows: (1) patients who reported a deterioration (a little, much or very much); (2) patients who reported ‘no change’ or ‘little improvement’; and (3) patients who reported ‘(very) much improvement’. The reason for including ‘little improvement’ in group 2 was the low number of patients in the category ‘no improvement’ (n = 6 at t38) and to create a more substantial reference group. Student–Newman–Keuls tests were performed to test the null hypotheses of ‘no differences between subgroups’. It was hypothesized that the CaReQoL Asthma very well reflects the GPE-scores at t38 (by showing significant differences in mean scores between the three groups at t38), thus proving the ability of this instrument to discriminate between patients with a different level of perceived effect, whereas changes in other scores (Δ t0–t38 AQLQ, ACQ, FEV_1_) are less likely to do so.

#### Responsiveness

Responsiveness is either defined as the effect of treatment, or as a measure of longitudinal validity defined by the correlation of changes in the instrument with changes in other measures [21]. In this study, the responsiveness of the CaReQoL Asthma, which aims to directly reflect the effects of pulmonary rehabilitation on several domains of health, was assessed in three steps. In step one, to investigate statistical significance of the longitudinal changes in care related aspects of health, the average scores on subsequent time points were compared. For the CaReQoL Asthma, the scores at t38 and t64 were compared by conducting one-sample paired t-tests, with the null hypotheses being ‘no difference between t38 and t64’. In step two, the CaReQoL Asthma scores at t38 were correlated with the change scores (Δ t0–t38) for other instruments, with the calculations and hypotheses being similar to those of the construct validity. In step three, the CaReQoL Asthma scores at t64 were correlated with the change scores for other instruments (Δ t0–t64), again with similar hypotheses as those formulated for the construct validity. See Table 3 for the hypothesized relationships.

#### Minimal important change

The minimal important change (MIC) was calculated for the CaReQoL Asthma at t38. The MIC is defined as the minimal change in a scale score that is considered to be important [20]. Firstly, three subgroups of the GPE were used (see "Criterion validity"). For each scale score, the average score of patients in group 2 (no or little improvement, see also "Criterion validity" for the consideration of combining these two groups) was subtracted from the average score of patients in group 3 (much or very much improvement). In addition, relevant changes or MICs of the AQLQ and ACQ scores (i.e. changes of at least 0.5 point) were also considered as external criteria for computing the MICs of the CaReQoL Asthma domains [17, 18].

## Results

### Descriptives and follow-up scores

The 83 patients who filled out the CaReQoL Asthma included 64 female (77%) and 19 male (23%) patients who had a mean age of 46 years (SD = 14). These 83 patients filled out the CaReQoL Asthma for the first time at t38 (6 months after rehabilitation), and 49 patients also filled out the CaReQol Asthma after one year (t64). The average scores for the CaReQoL Asthma, as well as for the other longitudinal measurements (AQLQ and ACQ and the predicted FEV_1_), are presented in Table 1. Highest scores of the CaReQoL Asthma at t38 were found for the subscales Knowledge about asthma (4.53) and Coping with asthma (4.23), followed by Physical functioning and Social functioning (3.76 and 3.62, respectively). On average, the pulmonary rehabilitation did not seem to affect the patients’ medication management as its mean score of 3.36 resembled the ‘neutral’ answering category of 3.0.

**Table 1:** Scores for CaReQoL asthma (t38, t64), AQLQ, ACQ and FEV_1_ (t0, t38, t64) (n = 83)

	Baseline t0	Follow-up t38	Follow-up t64
Mean scores:	Mean (95 CI)	Mean (95 CI)	Mean (95 CI)^#^
CaReQoL asthma:			
Total (1–5)	–	3.89 (3.73–4.06) (n = 77)	3.92 (3.68–4.16) (n = 47)
- Physical functioning (1–5)	–	3.76 (3.53–3.99) (n = 80)	3.66 (3.34–3.99) (n = 48)
- Social functioning (1–5)	–	3.62 (3.39–3.86) (n = 79)	3.70 (3.43–3.98) (n = 49)
- Coping with asthma (1–5)	–	4.23 (4.07–4.40) (n = 80)	4.23 (3.96–4.49) (n = 48)
- Knowledge about asthma (1–5)	–	4.53 (4.37–4.68) (n = 82)	4.57 (4.37–4.78) (n = 49)
- Medication (1–5)	–	3.36 (3.10–3.63) (n = 81)	3.41 (3.07–3.74) (n = 49)
Overall quality of life (1–5)	–	4.05 (3.82–4.29) (n = 80)	4.08 (3.76–4.40) (n = 49)
Global perceived effect (1–7)	–	5.35 (5.05–5.65) (n = 78)	5.29 (4.90–5.68) (n = 49)
AQLQ:			
Total	4.18 (3.96–4.40) (n = 83)	5.05 (4.78–5.32)^Δ^ (n = 67)	4.94 (4.65–5.24)* (n = 62)
- Symptoms	4.30 (4.07–4.53) (n = 83)	5.09 (4.80–5.39)^Δ^ (n = 67)	4.97 (4.67–5.26)* (n = 62)
- Activity limitations	3.67 (3.41–3.92) (n = 82)	4.66 (4.34–4.99)^Δ^ (n = 67)	4.54 (4.19–4.89)* (n = 62)
- Emotional functioning	5.08 (4.80–5.35) (n = 83)	5.94 (5.71–6.17)^Δ^ (n = 67)	5.87 (5.57–6.16) (n = 62)
- Environmental stimuli	4.12 (3.81–4.44) (n = 83)	4.88 (4.53–5.23)^Δ^ (n = 67)	4.82 (4.45–5.19) (n = 62)
ACQ	2.78 (2.56–2.99) (n = 81)	2.06 (1.78–2.34)^Δ^ (n = 66)	2.18 (1.87–2.49)* (n = 61)
FEV_1_	85.55 (80.64–90.45) (n = 82)	88.57 (82.65–94.49)^+^ (n = 61)	90.97 (83.85–98.08) (n = 47)

^Δ^Significant improvement compared to baseline (t0): paired t-tests, p < 0.001 (AQLQ: n = 67; ACQ: n = 64)

^+^Significant improvement compared to baseline (t0): paired t-tests, p < 0.01 (FEV_1_: n = 60)

*Statistically significant difference with t38, according to paired t-tests, p < 0.05 (ACQ; n = 52; AQLQ: n = 54)

^#^Differences between t38 and t64 not significant for CareQoL Asthma or GPE

### Missing data

The average percentage of item missing was 4% for the CaReQoL Asthma at t38. Sixty of the 83 patients (72%), provided complete data on all 26 CaReQoL items at t38. Of the 23 other respondents, 13 had one or two missing items, nine had three to 11 missing values, and one respondent had 21 missing values. Particularly one item of the domain Medication (item 23 about side effects of medication) showed missing values (16% missing compared to 7% or less for all other items). After 12 months (t64), 49 of the 62 participants in the total clinical study (79%) filled out the CaReQoL Asthma, due to incomplete follow-up as the CaReQoL Asthma was not administered to all patients at t64. Nevertheless, CaReQoL Asthma scores could be calculated for 77–82 of the 83 patients at t38, and for 47–49 patients at t64 (Table 1).

### Construct validity

The factor analyses on each subscale confirmed the structure of the CareQoL Asthma by showing high factor loadings for each item per subscale (see Table 2), resembling the findings of a previous study on the psychometric properties of the questionnaire [13]. Cronbach’s alpha for the five CaReQoL Asthma domains were: Physical functioning 0.95 (8 items), Social functioning 0.93 (4 items), Coping with asthma 0.85 (5 items), Knowledge about asthma 0.83 (3 items) and Medication 0.82 (3 items).

**Table 2:** Factor loadings and Cronbach’s alpha for subscales of the CareQoL Asthma (t38)

Factor		# items	Factor loadings (range)	Cronbach’s alpha
Physical functioning		8	0.72–0.90	0.95
Social functioning		4	0.85–0.88	0.93
Coping with asthma		5	0.65–0.78	0.85
Knowledge about asthma		3	0.71–0.91	0.83
Medication		3	0.72–0.78	0.82

Factor analyses were conducted for each subscale (Principal Component Analyses, with oblimin rotations; Eigenvalue > 1 and factor loadings > 0.40), n = 83

Table 3 shows the correlations between CaReQoL Asthma scores at t38 and the change scores of other measures between t0 and t38, together with the predefined hypotheses. Of the 45 correlations investigated, 38 (84%) were in accordance with the hypotheses. Only seven associations were either stronger (3×) or weaker (4×) than expected. For example, Social functioning showed moderate instead of weak correlations with AQLQ Symptoms and AQLQ Environmental stimuli (r = 0.48 and 0.42, respectively). On the other hand, Coping was much weaker associated with AQLQ Emotional functioning (r = 0.18) and ACQ (r = − 0.14) than expected.

**Table 3:** Construct validity and responsiveness of CaReQoL Asthma (t38 or t64)

CaReQoL domain (scores at t38 or t64)	Comparison instruments: change scores (∆ t0–t38 or ∆ t0–t64)	Expected correlation strength^#^ (direction)	Construct validity and responsiveness (t38)	Respon-siveness (t64)	Explanation/hypothesis
Physical functioning (8 items)	Δ AQLQ: Total score Symptoms Activity limitations Emotional functioning Environmental stimuli	0.40–0.59 (+) 0.40–0.59 (+) 0.40–0.59 (+) 0.00–0.39 (+) 0.00–0.39 (+)	0.48*** 0.53*** 0.39** 0.15 (n.s.) 0.29*	0.45** 0.44** 0.44** 0.30 (n.s.) 0.32*	Physical functioning is likely to be moderately related with changes in Total, Activity limitations and Symptoms scores, but not to other AQLQ scores
	Δ ACQ	0.40–0.59 (−)	− 0.52***	− 0.55***	Physical functioning is likely to be moderately related to a change in ACQ
	Δ FEV_1_	0.00–0.39 (+)	0.25 (n.s.)	0.15 (n.s.)	Physical functioning is expected to be weakly related to a change in FEV_1_
Social functioning (4 items)	Δ AQLQ: Total score Symptoms Activity limitations Emotional functioning Environmental stimuli	0.40–0.59 (+) 0.00–0.39 (+) 0.40–0.59 (+) 0.00–0.39 (+) 0.00–0.39 (+)	0.49*** 0.48*** 0.45*** 0.10 (n.s.) 0.42***	0.45** 0.40** 0.49** 0.14 (n.s.) 0.38*	Social functioning is likely to be moderately related to changes in the Total score and Activity limitations, but not to other AQLQ scores
	Δ ACQ	0.40–0.59 (−)	− 0.53***	− 0.43**	Social functioning is likely to be moderately related to a change in ACQ
	Δ FEV_1_	0.00–0.39 (+)	0.19 (n.s.)	0.07 (n.s.)	Social functioning is expected to be weakly related to a change in FEV_1_
Coping with asthma (5 items)	Δ AQLQ: Total score Symptoms Activity limitations Emotional functioning Environmental stimuli	0.00–0.39 (+) 0.00–0.39 (+) 0.00–0.39 (+) 0.40–0.59 (+) 0.00–0.39 (+)	0.16 (n.s.) 0.20 (n.s.) 0.09 (n.s.) 0.18 (n.s.) 0.06 (n.s.)	0.32* 0.24 (n.s.) 0.37* 0.07 (n.s.) 0.38*	Coping is likely to be moderately related to changes in Emotional function, but not to other AQLQ scores
	Δ ACQ	0.40–0.59 (−)	− 0.14 (n.s.)	− 0.46**	Coping is expected to be moderately related to a change in ACQ
	Δ FEV_1_	0.00–0.39 (+)	0.17 (n.s.)	0.01 (n.s.)	Coping is expected to be weakly related to a change in FEV_1_
Knowledge about asthma (3 items)	Δ AQLQ: Total score Δ ACQ Δ FEV_1_	0.00–0.39 (+) 0.00–0.39 (−) 0.00–0.39 (+)	0.17 (n.s.) − 0.15 (n.s.) 0.14 (n.s.)	0.36* − 0.23 (n.s.) 0.10 (n.s.)	Since there are no similar items on Knowledge about asthma in all other measures, correlations are expected to be very weak
Medication (3 items)	Δ AQLQ: Total score Symptoms Activity limitations Emotional functioning Environmental stimuli	0.00–0.39 (+) 0.40–0.59 (+) 0.00–0.39 (+) 0.00–0.39 (+) 0.00–0.39 (+)	0.38** 0.48*** 0.29* 0.02 (n.s.) 0.27*	0.23 (n.s.) 0.19 (n.s.) 0.28 (n.s.) -0.04 (n.s.) 0.28 (n.s.)	Medication is likely to be moderately related to changes in Symptoms, but not to other AQLQ scores
	Δ ACQ	0.40–0.59 (−)	− 0.47***	− 0.35*	Since 1 item of the ACQ concerns the use of medicine, a moderate correlation is expected
	Δ FEV_1_	0.00–0.39 (+)	0.08 (n.s.)	0.00 (n.s.)	Medication is expected to be weakly related to a change in the FEV_1_
Overall quality of life (QoL) (1 item)	Δ AQLQ: Total score Symptoms Activity limitations Emotional functioning Environmental stimuli	0.40–0.59 (+) 0.40–0.59 (+) 0.00–0.39 (+) 0.00–0.39 (+) 0.00–0.39 (+)	0.40** 0.41*** 0.30* 0.24 (n.s.) 0.37**	0.34* 0.27 (n.s.) 0.40* 0.05 (n.s.) 0.37*	The perceived effect on QoL is likely to be moderately related to changes in the Total and Symptoms scores, and weakly related to other AQLQ scores
	Δ ACQ	0.40–0.59 (−)	− 0.34**	− 0.45**	The overall quality of life is likely to be moderately related to a change in ACQ
	Δ FEV_1_	0.00-0.39 ( +)	0.15 (n.s.)	0.14 (n.s.)	The effect on QoL is expected to be weakly related to a change in the predicted FEV_1_
Global perceived effect (GPE) (1 item)	Δ AQLQ: Total score Symptoms Activity limitations Emotional functioning Environmental stimuli	0.40–0.59 (+) 0.40–0.59 (+) 0.00–0.39 (+) 0.00–0.39 (+) 0.00–0.39 (+)	0.46*** 0.47*** 0.43*** 0.09 (n.s.) 0.33**	0.46** 0.40** 0.51*** 0.18 (n.s.) 0.38*	This criterion variable GPE is expected to be moderately related to changes in the Total and Symptoms scores, and weakly related to changes in other AQLQ scores
	Δ ACQ	0.40–0.59 (−)	− 0.53***	− 0.50**	The GPE is expected to be moderately related to a change in ACQ
	Δ FEV_1_	0.00–0.39 ( +)	0.18 (n.s.)	0.20 (n.s.)	The GPE is expected to be weakly related to the FEV_1_

^#^Spearman’s correlation with the following classification: 0.00–0.39 ‘weak’, 0.40-0.59 ‘moderate’, 0.60–1.0 ‘strong’

Statistical level of significance: n.s. = non-significant; *p < 0.05; **p < 0.01; ***p < 0.001

### Responsiveness

The CaReQoL showed positive effects of the pulmonary rehabilitation (see Table 1), with an average total score of 3.89 on the 5-point scale (1–5) at t38. The effects of the rehabilitation appeared to remain stable over time as there were no significant differences in scores at 6 and 12 months (i.e. no changes between t38 and t64 according to paired t-tests). Similarly, the follow-up scores for the AQLQ, ACQ and FEV_1_ showed substantial and significant improvements after 6 months. These other scores also did not seem to change any further after 6 months, according to the overall scores and confidence intervals as displayed in Table 1, but paired t-tests for those who responded to all follow-up measurements indicated that the AQLQ total, AQLQ Symptom and AQLQ Activity limitations scores as well as the ACQ did significantly change and somewhat diminished between 6 and 12 months (AQLQ: n = 54, ACQ: n = 52; p < 0.05).

Table 3 shows the correlations between CaReQoL Asthma scores at t38 and t64 and change scores of all other instruments (differences between t0–t38 and t0–t64, respectively). Similar to the construct validity and as hypothesized, the scales on Physical functioning, Social functioning, and the 1-item measuring the global perceived effect (GPE) and the quality of life (QoL) were moderately and significantly associated with changes in the AQLQ scores (in particular: Total, Symptoms and Activity limitations) and the ACQ. These Spearman’s coefficients ranged between 0.40 and 0.59. Weak and often non-significant correlations were found for the other CaReQoL scales and the predicted FEV_1_.

At t64, both Coping with asthma and Knowledge about asthma show more responsive, higher and significant correlations with changes in AQLQ or ACQ scores. In particular Coping with asthma, which was related to AQLQ and ACQ scores at t64, whereas these associations did not exist at t38. Medication on the other hand, showed less significant correlations at t64 compared to t38.

### Criterion validity

Answers on the global perceived effect (GPE) of the pulmonary rehabilitation at t38 showed a deterioration in eight patients (group 1), no or a little improvement in 25 patients (group 2), and much or very much improvement in 45 patients (group 3). Five patients did not fill out the GPE and were missing. Table 4 presents the scores of the CaReQoL Asthma (total and mean scale scores, and overall quality of life) and change scores (t0–t38) for the AQLQ, ACQ and FEV_1_ for each GPE- group.

**Table 4:** Criterion validity of CaReQoL asthma and AQLQ, ACQ and FEV_1_ (t38)

Mean scores (t38)	Global perceived effect (t38):		
	Improvement^#^	No or little improvement	Deterioration^^^
CaReQoL asthma	(n = 45)	(n = 25)	(n = 8)
Total score**	4.35***	3.51**	2.93***
- Physical functioning	4.37***	3.28**	2.46***
- Social functioning	4.24***	3.14**	2.07***
- Coping with asthma	4.47	4.12	3.66*
- Knowledge about asthma	4.66	4.42	4.46
- Medication	3.98***	2.64	2.38***
Overall QoL	4.56**	3.67*	2.40***
Mean change scores (∆ t0–t38):			
∆ AQLQ:	(n = 38)	(n = 18)	(n = 6)
Total score	1.12**	0.39	0.02*
- Symptoms	1.15**	0.21	− 0.10*
- Activity limitations	1.26*	0.49	− 0.15*
- Emotional functioning	0.90	0.55	0.70
- Environmental stimuli	0.92	0.50	0.00
∆ ACQ	− 1.06** (n = 37)	− 0.11 (n = 17)	0.30* (n = 5)
∆ FEV_1_	4.59 (n = 34)	3.55 (n = 17)	− 2.22 (n = 5)

^#^Much or very much improvement according to GPE

^^^Little/ much/ very much deterioration according to GPE

Level of significance of differences between subsequent subgroups (column1; better vs. the same, column2; the same vs. deterioration, column3; deterioration vs. better; SNK-tests): *p < 0.05; **p < 0.01; ***p < 0.001

Almost all scores of the CaReQoL Asthma showed significant differences between two or three of the GPE-groups, except Coping with asthma and Knowledge about asthma. Other instruments showed few significant differences between the GPE-groups; only some changes in AQLQ scores (Total, Symptoms and Activity limitations) and the ACQ discriminated between some of the GPE-groups.

### Minimal important change

The minimal important changes (MICs) for the CaReQoL Asthma scales at t38 are: Physical functioning 1.09, Social functioning 1.10, Coping with asthma 0.35, Knowledge about asthma 0.24, and Medication 1.34 (Table 5). Unfortunately, no additional analyses could be conducted to assess MICs in relation to relevant changes between t0 and t38 in AQLQ (an increase of at least 0.5) or ACQ (a decrease of at least 0.5), as external criteria, due to small numbers of patients with ‘no relevant change’ (n = 8 and n = 7 at t38; n = 9 and n = 12 at t64, respectively).

**Table 5:** Minimal important change (MIC) for CaReQoL asthma scores (t38)

	CaReQoL Asthma: mean scores at t38					
	Physical functioning	Social functioning	Coping with asthma	Knowledge about asthma	Medication	Total
Global perceived effect (t38):						
Much or very much improvement (n = 45)	4.37	4.24	4.47	4.66	3.98	4.35
No or little improvement (n = 25)	3.28	3.14	4.12	4.42	2.64	3.51
Minimal important change (MIC)*	1.09	1.10	0.35	0.24	1.34	0.84

*Difference in the CaReQoL Asthma score between ‘(very) much improvement’ and ‘no or little improvement’

## Discussion

This paper describes the psychometric properties and validity of the CaReQoL Asthma, a self-report questionnaire specifically designed for evaluating the perceived outcomes of pulmonary rehabilitation in patients with severe asthma. The instrument aims to comprehensively describe the Care Related Quality of Life outcomes regarding the treatment goals of pulmonary rehabilitation that are important to patients [13]. Validation was based on longitudinal data collected among 83 patients with severe refractory asthma who took part in an observational clinical study on the effectiveness of pulmonary rehabilitation in two tertiary asthma clinics in Switzerland and The Netherlands [15]. This study confirms that the relatively short CaReQoL Asthma (26 items) is a comprehensive, valid, reliable and responsive questionnaire for assessing relevant outcomes of pulmonary rehabilitation in patients with severe refractory asthma.

### Good psychometric properties

Findings show that the CaReQoL Asthma has a good construct validity and that it could reliably measure the long-term impact of multidisciplinary and personalized rehabilitation programs on the patients’ lives, regarding five domains: Physical functioning (Cronbach’s alpha = 0.95), Social functioning (alpha = 0.93), Coping with asthma (alpha = 0.85), Knowledge about asthma (alpha = 0.83) and Medication (0.82).

The new instrument even appears to assess outcomes of rehabilitation that are only partly or not at all assessed by the other well-known disease-specific instruments AQLQ and ACQ that were used as primary and secondary outcome of the total clinical study [15]. Particularly Coping with asthma and Knowledge about asthma turned out to be unique domains of the CaReQoL Asthma as these scores showed substantial effects of the pulmonary rehabilitation but were only weakly correlated with the two other questionnaires (AQLQ and ACQ).

Furthermore, the convergent and discriminant/divergent validity and the responsiveness of the CaReQoL Asthma, as compared to changes in AQLQ, ACQ and FEV_1_ scores, were in line with our expectations (i.e. 84% conform pre-defined hypotheses). The very low and insignificant correlations with the FEV_1_ underline the hypotheses that this clinical measure does not reflect the patients’ self-reported health.

Moreover, the CaReQoL Asthma showed good criterion validity and discriminant scores in relation to the global perceived effect (GPE) as a criterion variable. The CaReQoL domain scores discriminated better than other measures between three levels of the perceived effect at 6 months post-rehabilitation, except for the domain Knowledge about asthma which appeared to be unrelated to the perceived effect.

The minimum important change (MIC) for the CaReQoL Asthma total scale is estimated at 0.84 and varies per domain, from 0.24 (Knowledge about Asthma) to 1.34 (Medication). However, these MICs are likely to be somewhat overestimated as real minimal differences could not be established in this study because of very low number of patients reporting ‘no improvement’. While this GPE-reference group had to be merged with ‘little improvement’ to create a more substantial subgroup, this might have led to an overrating of the MICs. The estimated MICs for the CaReQoL Asthma resemble the findings of a previous study showing minimal important differences from 0.21 to 1.16 per domain [13]. Thus, an overall MIC of 0.8 and MICs between 0.2 to 1.2 for the five domains seem to be realistic.

### Coping and knowledge

The CaReQoL domains Coping with asthma and Knowledge about asthma had the highest average scores at 6 and 12 months post-rehabilitation. These high scores reflect major and significant contributions of the rehabilitation trajectory to the patients' coping skills and knowledge about asthma. But these two domains appeared to be the least responsive to change, compared to other measures, and also had the lowest and least reliable MICs (0.35 for Coping and 0.24 for Knowledge) because of mainly non-significant differences between the GPE-groups (i.e. the criterion measure).

Interestingly, the increased coping and knowledge of patients due to the rehabilitation (according to the CaReQoL) seem to have more persistent long-term effects on the quality of life (AQLQ) and asthma control (ACQ), as correlations between these outcomes increased over time. Particularly coping was related to an increase in asthma control after one year. Apparently, increased coping skills and knowledge on asthma may persist over time and could still reinforce or prolong the effects of pulmonary rehabilitation by contributing to the patients’ quality of life in the long run. This is in line with literature on various interventions aimed to improve asthma knowledge, self-management, medication adherence and inhalation techniques (e.g. objective monitoring, feedback, education or training and group learning) which in turn may result in an increased quality of life and clinical outcomes [22, 23]. But the underlying mechanism of the effects of pulmonary rehabilitation and the mediating effects of increased knowledge and coping skills on the self-management, health outcomes and quality of life of patients with severe asthma still needs further investigation [22, 23].

### Long-term effects

Altogether, the follow-up measurements with the CaReQoL Asthma showed substantial and enduring effects following the pulmonary rehabilitation on the patients’ quality of life and other health outcomes, still present after 12 months. In general, the follow-up measurements at 6 and 12 months showed a substantial improvement to the patients’ knowledge and coping with asthma after rehabilitation, followed by an increased physical and social functioning, but a rather weak change in their medication management. These results are in line with the previously reported findings of the total clinical study [15] and other studies on the benefits of personalized pulmonary rehabilitation programs [5, 24, 25]. Additionally, the clinical study showed the strongest effects on quality of life and asthma control at the end of the rehabilitation trajectory (after 12 weeks), and these effects were larger and more sustainable in the high-altitude group than in patients who followed a rehabilitation program at sea-level [15].

### Study limitations

The present study has some limitations. Firstly, the number of patients included in this study is low. A total of 83 patients were included at t38, which is normally too small for exploring internal consistency and construct validity [20]. However, the factor analyses as well as other psychometric analyses were also conducted in a previous study (published in dutch), consisting of 195 respondents and showing similar findings [13]. In addition, for testing a priori hypothesis, as is done for construct validity and responsiveness, a total of 50 respondents seems adequate, which is met for measurements at t38 [20]. Nevertheless, conclusions should be drawn with caution and replication is warranted. Secondly, the numbers of listwise missings are high: of the 83 who filled out the CaReQoL Asthma at t38, 60 had no missing items at t38 and only 38 patients also participated and fully completed the questionnaires at t64. This loss to follow-up was partly due to incomplete data collection as not all study participants in the total clinical study additionally received a CaReQoL Asthma at t38 and t64. But there did not seem to be selection bias since there were no differences in demographic or clinical characteristics between those who dropped out and those who completed the follow-up of the total clinical study [15]. Another limitation for validating the CaReQoL Asthma concerns the few and long-term measurement points at which our instrument was applied, given the focus of the clinical study [15]. Evaluations during and at the end of the pulmonary rehabilitation, for example half-way and after 12 weeks, would have yielded better data to study the responsiveness and criterion validity of the instrument. These measurement points are also recommended for implementing the instrument in clinical practice for the purposes of shared decision making, monitoring and quality improvement [26]. Finally, as the CareQol Asthma is a direct measure requiring respondents to report *change* in functioning as compared to baseline, baseline measures were effectively obtained retrospectively and therefore susceptible to recall bias [27]. However, the obvious alternative would have been an indirect measure of change where respondents only report current level of functioning at baseline (t0) and follow-up (t38 and t64) and change is calculated as the difference between baseline and follow-up. In such a design, the baseline measure would be susceptible to response shift, i.e., the phenomenon that internal standards of severity of symptoms change over time [28]. Although it is commonly assumed that recall bias is a bigger problem than response shift, empirical results appear to be mixed [27, 28],

### Future research

Future studies with the CaReQoL Asthma could reveal more specific effects of the pulmonary rehabilitation in patients with severe asthma over time, including increased knowledge and coping skills. Furthermore, future research may yield more insight into the underlying mechanisms that either enhance or hamper disease management and shared decision making on the personalized treatment, not only for severe asthma but perhaps also for other respiratory disorders (i.e. mild asthma and COPD) and other treatment modalities (regular treatment or specific disciplines, e.g. physiotherapy). In addition, future studies should aim to replicate the psychometric properties of the CareQoL Athma in bigger samples.

### Conclusions

Our findings show good properties for the construct validity (internal consistency, convergent and divergent validity), responsiveness and criterion validity of the CaReQoL Asthma at 6 months post-rehabilitation. This instrument is the first PROM that was specifically designed for measuring outcomes of pulmonary rehabilitation in patients with severe asthma. It has been developed in close collaboration with patients and fully describes the impact of the pulmonary rehabilitation on life domains which are important to patients. Moreover, our results suggest that this instrument may be regarded as a unique and complementary instrument as it does not measure exactly the same constructs as the AQLQ and ACQ and it comprises specific domains on knowledge and coping with asthma which are not covered by these instruments. In order to further substantiate the reliability and validity of the CaReQoL Asthma, as well as to monitor outcomes of pulmonary rehabilitation in patients with severe asthma, we recommend to use the CaReQoL Asthma in addition to other disease specific instruments.

## Data Availability

All relevant data is presented in the manuscript and associated tables. The data that support the findings of this study are available from UMC Utrecht; however, restrictions apply to the availability of these data, which were used under license for the current study and are not publicly available.

## Appendix

### CaReQoL asthma

#### Questionnaire on the ‘care related quality of life’ in persons with severe asthma

Please answer the following propositions about the effects that you might experience after your recent pulmonary rehabilitation. The questions are about your opinion and the perceived effects during the past two weeks.

Add your response to each question starting with ‘*Due to the pulmonary rehabilitation…*’.

You may choose ‘not applicable’ (N.A.) when the question is not applicable to you, or when a change or effect is not related to your asthma.

**Table:**

	Physical functioning Due to the pulmonary rehabilitation…	Totally disagree	Partly disagree	Neither agree, nor disagree	Partly agree	Totally agree	N.A
1	…I am better able to climb stairs	❏	❏	❏	❏	❏	❏
2	…I can walk for a longer time	❏	❏	❏	❏	❏	❏
3	…I am better able to play sports or to do physical exercises or workouts	❏	❏	❏	❏	❏	❏
4	…I am in better shape (more physically fit)	❏	❏	❏	❏	❏	❏
5	…I am less tired	❏	❏	❏	❏	❏	❏
6	…I suffer less from a shortness of breath	❏	❏	❏	❏	❏	❏
7	…my health status is more stable	❏	❏	❏	❏	❏	❏
8	…I have less asthma attacks	❏	❏	❏	❏	❏	❏

**Table:**

	Social functioning Due to the pulmonary rehabilitation…	Totally disagree	Partly disagree	Neither agree, nor disagree	Partly agree	Totally agree	N.A
9	…I can better conduct my daily activities (such as work, study or housekeeping)	❏	❏	❏	❏	❏	❏
10	…I do things more often spontaneously	❏	❏	❏	❏	❏	❏
11	…getting out and about with others (such as pleasure trips, visiting, going out) is easier for me	❏	❏	❏	❏	❏	❏
12	…doing things in and around the house (such as in-house jobs or shopping) is easier for me	❏	❏	❏	❏	❏	❏

**Table:**

	Coping with asthma Due to the pulmonary rehabilitation…	Totally disagree	Partly disagree	Neither agree, nor disagree	Partly agree	Totally agree	N.A
13	…I am in a better mood	❏	❏	❏	❏	❏	❏
14	…I can cope better with my asthma	❏	❏	❏	❏	❏	❏
15	…I have better control over my asthma (mastering my asthma)	❏	❏	❏	❏	❏	❏
16	…I am better able to make my own choices	❏	❏	❏	❏	❏	❏
17	…I am more aware of my own limits	❏	❏	❏	❏	❏	❏

**Table:**

	Knowledge about asthma Due to the pulmonary rehabilitation…	Totally disagree	Partly disagree	Neither agree, nor disagree	Partly agree	Totally agree	N.A
18	…I have more knowledge of my asthma (and I can explain it better to others)	❏	❏	❏	❏	❏	❏
19	…I have more knowledge of my asthma medication	❏	❏	❏	❏	❏	❏
20	…I know better what to do in case of a shortness of breath	❏	❏	❏	❏	❏	❏

**Table:**

	Medication Due to the pulmonary rehabilitation…	Totally disagree	Partly disagree	Neither agree, nor disagree	Partly agree	Totally agree	N.A
21	…I need less medication	❏	❏	❏	❏	❏	❏
22	…I am better adjusted to my medication	❏	❏	❏	❏	❏	❏
23	…I experience less side-effects of my medication	❏	❏	❏	❏	❏	❏

	Overall Due to the pulmonary rehabilitation…	Totally disagree	Partly disagree	Neither agree, nor disagree	Partly agree	Totally agree	N.A
24	…my quality of life has improved	❏	❏	❏	❏	❏	❏

25 How is your health now, compared to your health before the pulmonary rehabilitation?
  - Very much improvement
  - Much improvement
  - Little improvement
  - No change
  - Little deterioration
  - Much deterioration
  - Very much deterioration
  - I don’t know/not applicable
26 What is your age?
  ____ years.

## Abbreviations

CaReQoL: Care Related Quality of Life in Asthma
PROM: Patient reported outcome measure
MIC: Minimal important change
AQLQ: Asthma Quality of Life Questionnaire
ACQ: Asthma Control Questionnaire
FEV_1_: Forced expiratory volume in one second
GPE: Global perceived effect
95CI: 95% Confidence interval
SD: Standard deviation
SNK: Student–Newman–Keuls test
